# Reshaping Egyptian funerary ritual in colonized Nubia? Organic characterization of unguents from mortuary contexts of the New Kingdom (c. 1550–1070 BCE)

**DOI:** 10.1007/s12520-023-01769-6

**Published:** 2023-05-08

**Authors:** Rennan Lemos, Kate Fulcher, Ikhlas Abdllatief, Ludmila Werkström, Emma Hocker

**Affiliations:** 1 Department of Archaeology, University of Cambridge, Downing Street, Cambridge, CB23DZ UK; 2 Department of Scientific Research, British Museum, London, UK; 3 National Corporation for Antiquities and Museums, Khartoum, Sudan; 4 Gustavianum, Uppsala University, Uppsala, Sweden

**Keywords:** Nubia, Sudan, Egypt, Colonialism, Mortuary practices, Canopic jars, Bitumen

## Abstract

Samples taken from the canopic jars of Djehutyhotep, chief of Tehkhet (Debeira), Lower Nubia, and local versions of Egyptian canopic jars from Sai, Upper Nubia, suggest that the materials used for mortuary ritual unguents in Nubia may have differed from those used in Egypt. Nubian samples consisted of plant gum and bitumen, whereas those from Egypt conformed to the standardizing black resinous liquid recipe used for mummification and other funerary rituals. However, there may be time frame issues to be considered as most samples analyzed from Egypt date to later periods. A standard black funerary liquid was used at Amara West, Upper Nubia, probably poured over a wrapped body, which might suggest that the gum and bitumen mixture was reserved for filling canopic jars, perhaps indicating that the use of canopic jars in Nubia differed from their use in Egypt. Evidence from the canopic jars of Djehutyhotep, local versions of canopic jars from Sai, and the sample from Amara West also indicate a source of bitumen that was not the Dead Sea, which was the main (although not only) source used in Egypt. The new results from the analysis of the Djehutyhotep canopic jars and previously published results from Sai point towards alternative ritual practices associated with local conceptions and uses of canopic jars in colonized Nubia. These samples and data from Amara West further reveal that the bitumen used in mortuary contexts in Nubia originated elsewhere than bitumen used in Egypt, which might have implications for our understanding of colonized Nubia as part of other trade networks independently from Egypt.

## Introduction

**Fig. 1:**
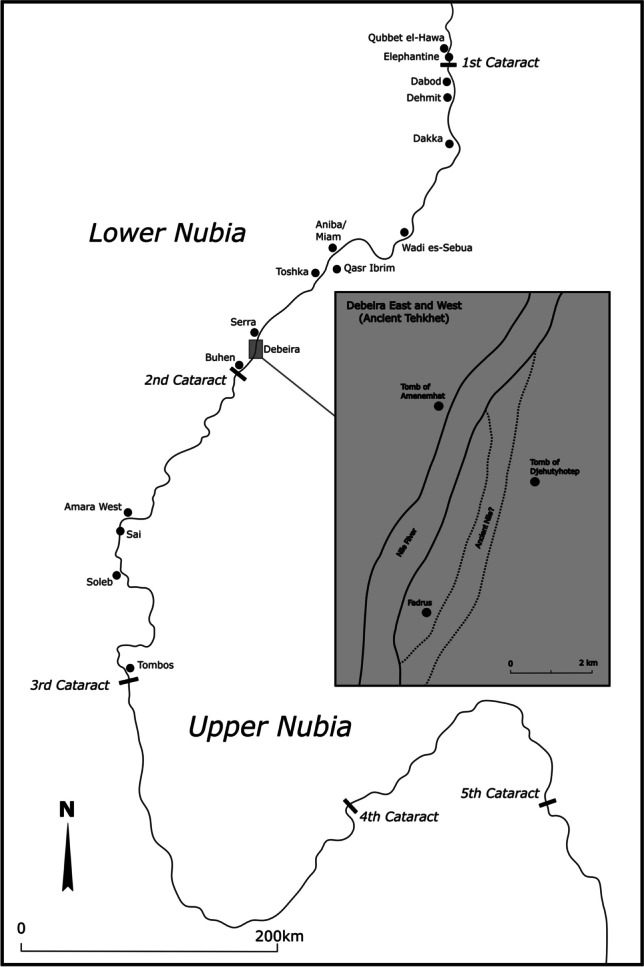
Map of colonized Nubia during the New Kingdom showing the sites mentioned in the text. Toponyms refer to modern villages. The approximate location of the tombs of brothers Djehutyhotep and Amenemhat is highlighted (22° 8′ 17.127", 31° 23′ 18.5784"). The landscape is now lost under the waters of Lake Nubia. Map by R. Lemos

During the New Kingdom (1550–1070 BCE), the ancient Egyptians colonized the whole extent of Nubia, the area comprising southern Egypt from Aswan to the fifth cataract of the Nile in Sudan (Fig. 1). During this period, several objects of the Egyptian “burial system” (Smith 1992) reached Nubia, substituting, to a large extent, previous indigenous material culture while materializing the Egyptian colonial presence in the region (Thill 1996; Lemos 2020). This has been traditionally understood as evidence for the “Egyptianization” of Nubians (Reisner 1910: 340–342; Säve-Söderbergh 1941: 129–135; Kemp 1978: 34–35; Török 2009: 264). However, recent work on Egyptian-style material culture has moved away from such problematic perspectives, revealing the complex dynamics behind the adoption of these objects in local contexts in colonized Nubia. On the one hand, imported Egyptian objects formed part of the burials of wealthy individuals who wished to create and display connections with Egypt. On the other hand, locally adapted versions of imported Egyptian material culture, or local versions of these objects, attest to creative engagements with the colonizer’s patterns resulting in material innovation (Smith 2021; Smith and Buzon 2017: 621–624; Lemos 2021: 262; Budka 2021: 123–124). However, knowledge of specific cultural practices surrounding the adoption of Egyptian-style material culture in mortuary contexts in colonized Nubia beyond typologies remains limited, especially concerning religion and mortuary rituals. While abundant textual evidence from Egypt provides details regarding the steps of mortuary rituals and the function of specific objects that comprised mortuary assemblages, this information should not be simply transplanted to New Kingdom Nubia, which risks obscuring socio-cultural diversity in a context of colonization (Lemos 2023).

Macro-scale approaches to material culture typologies and adapted versions of standardizing Egyptian types widespread through colonized Nubia only allow us to understand broadly the social contexts created by these material assemblages. This includes questions of access to restricted, imported objects and their newly attributed social roles following local adaptation and material transformation to create social distinction (Lemos 2021; 2023; Lemos and Budka 2021). Macro-scale approaches based on object typologies and styles pose clear limitations to understanding practices attached to objects in the absence of texts. On the contrary, micro-scale approaches such as residue analysis can shed light on aspects of cultural practices associated with Egyptian-style objects in colonized Nubia. Thus far, only a few samples taken from mortuary objects have been analyzed at a micro-scale level for the New Kingdom colonial Nubia. These are progressively revealing diverse aspects of ritual practices connected to specific types of grave goods (Fulcher et al. 2020; Fulcher and Budka 2020).

In light of previous analyses of resinous liquids used in mortuary rituals in New Kingdom colonial Nubia, this paper presents the results of organic residue analysis of samples taken from three canopic jars of chief Djehutyhotep—an indigenous leader who benefited from the Egyptian colonial exploitation of Nubia in the reigns of Hatshepsut and Thutmose III (1473–1458 BCE) (Moss 1950: 41–42; Thabit 1957: 80; Säve-Söderbergh 1960: 29–30; 1991: 187; Säve-Söderbergh and Troy 1991: 204). Djehutyhotep, also known by the Nubian name Paitsy, was part of a local ruling family with deep ties to the Egyptian colonial administration dating back to previous reigns. The three canopic jars analyzed here, originally op were found in the tomb of his brother and successor Amenemhat (Fig. 1).

Djehutyhotep/Paitsy is known from inscriptions related to his family coming from various parts of Lower Nubia in the New Kingdom colonial period, namely Qubbet el-Hawa (tomb of his relative Senmose), Elephantine, Qasr Ibrim, Buhen, Serra, and Debeira East and West (Säve-Söderbergh and Troy 1991: 191–204). These texts allow us to reconstruct a long line of indigenous elite individuals and their connections with the Egyptian colonial administration of Lower Nubia at least two generations before Djehutyhotep. Both his grandfather, Teti (also referred to in textual sources as Djawia, his Nubian name), and his father, Ruiu, bore the title “great (= chief) of Tehkhet.” Djehutyhotep inherited the same title and then passed it on to his younger brother and successor, Amenemhat. Djehutyhotep and Amenemhat also bore the title of “scribe,” which is evidence for their direct role in the administration of the Egyptian colony and, potentially for their Egyptian education (Säve-Söderbergh 1991). Fragmentary textual evidence from Serra mentions a chief of Tehkhet named Ipi, who was granted land by the Viceroy of Kush Heqanakht, active in the reign of Ramses II (1276–1259 BCE) (Säve-Söderbergh and Troy 1991: 204; Török 2009: 174). Evidence for Djehutyhotep’s family demonstrates the longevity of the collaboration between Egyptian colonizers and Indigenous elites operating the colonial system of exploitation of Nubia throughout the New Kingdom (Müller 2013: 432–434). For instance, Djehutyhotep himself was involved in the Egyptian colonial exploitation of Nubian gold (Davies 2020: 200–201). This collaboration with Egyptian colonizers resulted, on the one hand, in a few individuals materially benefitting from foreign colonization, mostly expressed as wealthy burials, while, on the other hand, most of the population experienced material scarcity (Lemos forthcoming), resulting in metamorphosed objects and local versions of imposed material patterns (Lemos 2023: 9–10) or alternative uses of dominant material patterns in contexts where structural limitations imposed major challenges (Lemos 2021: 262–266).

A result of Djehutyhotep’s family collaboration with, and participation in, the Egyptian colonization of Nubia were the monumental, decorated rock-cut tombs owned by its members: Senmose at Qubbet el-Hawa in Aswan (de Morgan et al. 1894; Edel 1963), Djehutyhotep/Paitsy at Debeira East (Thabit 1957; Wild 1959; Säve-Söderbergh 1960), and Amenemhat at Debeira West (Säve-Söderbergh and Troy 1991). Other rock-cut tombs in Debeira might have belonged to family members or associates (Sherif 1960). In the New Kingdom, members of the Egyptian elite owned monumental decorated rock-cut tombs, especially at places such as Thebes (Kampp 1996) and Saqqara (Staring 2022). On the contrary, rock-cut tombs were extremely rare in the New Kingdom colonial Nubia, with only a handful of tombs known to date, which are still to be fully understood (Spence 2019: 546). In the 18th dynasty, the few decorated rock-cut tombs of Nubian elite individuals connected to the Egyptian colonial system include scenes depicting those individuals as Egyptians, even when Egyptians themselves would depict local Nubian chiefs in their tomb scenes as foreigners bearing all material identity markers that would characterize them as Nubians. This is the case of Hekanefer, who owned a rock-cut tomb at Toshka, where he was depicted as an Egyptian (Simpson 1963: 9), while he was represented as a Nubian in the tomb of Viceroy of Kush Huy at Thebes (Davies and Gardiner 1926: plate XXVIII; van Pelt 2013: 534–538; Smith 2015).

**Fig. 2:**
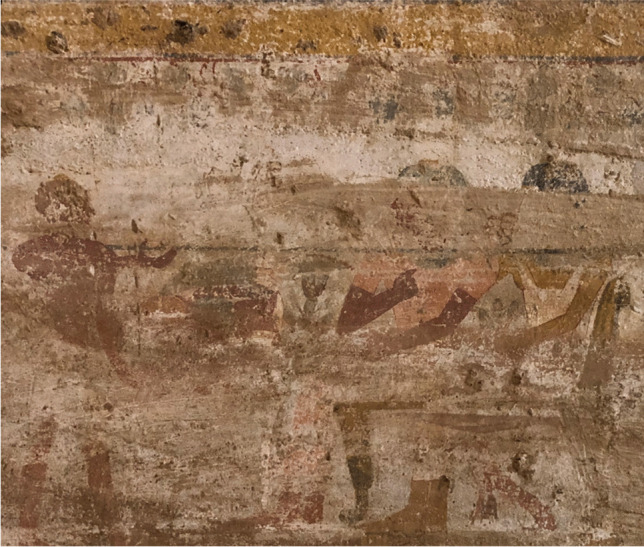
Part of the banquet scene on the north wall of the large chamber in the tomb of Djehutyhotep, in which he and his wife Tenetnebu are seated facing an offering table. On top of the offering table, there are a bundle of onions, meat, and flowers. In front of the offering table, a standing man offers a cup to the couple (photo by R. Lemos, courtesy of the Sudan National Museum)

The New Kingdom tomb of Djehutyhotep (mid-18th dynasty) was first recognized in the 1930s and fully excavated in the 1950s following modern plundering (Arkell 1950: 24–25). The tomb was cut in a sandstone hill east of Debeira and comprised a large decorated chamber containing wall paintings depicting Djehutyhotep as an Egyptian official, for instance, a banquet scene depicting him and his wife in a typically Egyptian way (Fig. 2). This and other scenes from the tomb have been interpreted as conveying religious meanings, which would have worked as the expression of their acculturation or “Egyptianization” (Török 2009: 269), even if differences in artistic composition and arrangement can be identified in comparison with contemporary Egyptian tombs at Thebes. Other indigenous chiefs of Lower Nubia have been represented as Egyptians in their tombs; e.g., Hekanefer, chief of Miam. In his tomb at Toshka (Aniba area), Hekanefer was also depicted wearing Egyptian garments, while he appears in the tomb of the Viceroy of Kush Huy at Thebes (TT40) dressed as a Nubian. Evidence from the tombs of Djehutyhotep, Hekanefer, and Amenemhat is evidence for their social standing resulting from their collaborations with the Egyptian colonial exploitation of Nubia. Differences in ritual practices understood from micro-analyses of specific Egyptian-style objects from these tombs allow us to move away from problematic perceptions of “acculturation” towards an understanding of the social impact of cultural contacts in the New Kingdom colonial period.

In the tomb of Djehutyhotep, the large chamber decorated with wall paintings led to a shrine containing the Egyptian-style statues of Djehutyhotep, his wife, and his parents, and another smaller chamber where a deep shaft leading to underground chambers was located (Thabit 1957: 82–84). Evidence from wooden coffins suggests that the tomb was reused from the late 18th dynasty to the 20th dynasty (Taylor 2017). Objects that were part of the original burial assemblage of Djehutyhotep were also removed from his tomb soon after his burial and ended up in the tomb of his brother Amenemhat in Debeira West (late 18th dynasty). These include two broad ceramic flasks inscribed for “scribe Djehutyhotep” (Holthoer 1977: plate 33); two scribe’s palettes, one bearing the text “scribe Paitsy” and the other “great of Tehkhet, Paitsy” (Säve-Söderbergh and Troy 1991: 202–203); and a complete set of four inscribed canopic jars for Paitsy (and a variation of the same name, Paitsy-her), three of which are discussed in this paper (Säve-Söderbergh and Troy 1991: 203). It is difficult to determine whether objects from the original burial assemblage of Djehutyhotep were removed from his tomb to be used as grave goods by his successor Amenemhat or if they were reused and deposited in the tomb of Amenemhat as part of later New Kingdom burial assemblages (cf. Säve-Söderbergh and Troy 1991: 188). Nevertheless, these objects shed light on local experiences of colonization in New Kingdom Nubia, especially in comparison with similar burial contexts and material culture of the same period (Lemos 2020).

In a context of widespread substitution of indigenous material patterns for objects materializing the Egyptian colonization in Nubia during the New Kingdom (Lemos 2020), macro-scale analyses of Egyptian-style material culture can shed light on different social experiences of colonization through objects that shaped different perceptions of culture (e.g., elites versus non-elites). For instance, the material culture from mortuary contexts allows us to distinguish adaptations and local versions of Egyptian objects that would potentially have entailed alternative practices both in comparison with Egypt and across different groups within Nubia (Lemos 2020: 20). The highest local elites, including Djehutyhotep, Amenemhat, or Hekanefer, owned rare decorated rock-cut tombs and imported objects from Egypt, but would their practice of Egyptian-style tombs and objects match Egyptian mortuary practices of the New Kingdom? Other local elites had access to restricted Egyptian-style objects, but they experienced, to a certain degree, material scarcity, which led to adaptations resulting in reshaping objects in essentially local ways. These local objects would not fit Egyptian expectations of mortuary objects and probably indicate variations in religious conceptions and practices (Lemos 2021: 262). Our knowledge of non-elite worldviews and practices remains particularly limited in the context of material scarcity resulting in a majority of tombs with no burial goods or very few items (Spence 2019: 557).

In a context of complex cultural interactions shaped by different social experiences of colonization and taking into account that Egyptians themselves saw individuals like Djehutyhotep fully as Nubians, micro-analyzing their material culture might reveal that different practices took place around their adoption of Egyptian-style material culture. This would allow us to further move away from homogenizing perspectives, and at the same time, it adds an extra layer of complexity to our understanding of different experiences of Egyptian imposed culture dictated by different social experiences of the material world in a colonial situation.

The material culture associated with Djehutyhotep has never been properly investigated and published. This is the first time his canopic jars have been studied. Despite the fact they were retrieved from Amenamhat’s tomb across the river, these jars work as evidence for indigenous elite religious conceptions and practices in the Nubian colony. These elites displayed clear affinities with Egyptian culture through their decorated tombs and imported Egyptian objects. However, would their practice have remained local? The use of Nubian names by individuals such as Djehutyhotep and Teti would point towards the maintenance of local cultural connections in a colonial context dominated by Egyptian cultural patterns, which is also suggested by the preservation of Nubian foodways in a context of widespread adoption of Egyptian pottery (Smith 2003a). Investigating the canopic jars of Djehutyhotep on a micro-scale would shed light on aspects of cultural and religious practices among the highest colonial elites who desired to express deep cultural affinities with Egypt.

## The canopic jars of Djehutyhotep/Paitsy, chief of Tehkhet

Canopic jars appeared in Egypt in the Old Kingdom to contain the internal organs and viscera removed from the mummified body of the deceased. These jars could be placed in chests and were no longer used by the Roman Period. In the New Kingdom, canopic jars were tall, broad vessels made of various stone types and pottery, with variation within this period. In the 18th dynasty, lids were shaped like human heads, and jars were inscribed with a standard inscription mentioning the four sons of Horus—Imsety, Hapy, Duamutef and Qebehsenuef—protectors of the organs stored inside. Animal-shaped lids for Hapy, Duamutef, and Qebehsenuef started to become common in the Ramesside Period (Ikram and Dodson 1998: 276–288).

**Fig. 3:**
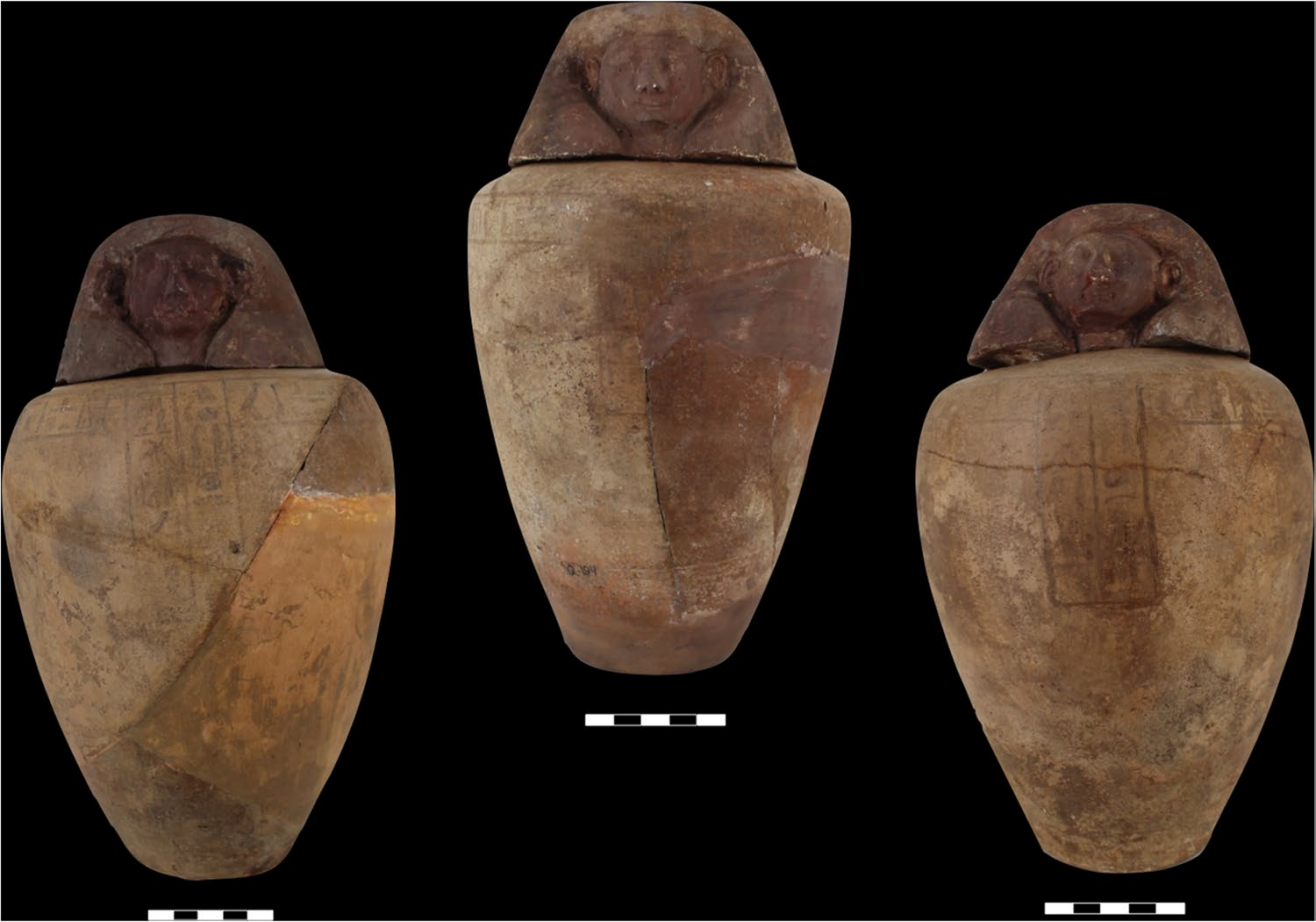
Three of the set of four 18th dynasty canopic jars of Djehutyhotep, chief of Tehkhet (photos by R. Lemos, courtesy of the Gustavianum Museum, Uppsala University). From left to right: Q10:42 (H 24 cm), Q10:194 (H 25 cm), and Q10:107 (H 25 cm). The fourth canopic jar of Djehutyhotep (Q10:148) is kept in the Sudan National Museum in Khartoum (SNM 13,184) and remains to be analyzed. Inscriptions are highly faded or damaged, which makes it difficult to reconstruct them fully. Jar Q10:42 (Duamutef) bears an offering formula to Anubis; Q10:194 (illegible, probably Imsety), bears an offering to Osiris; and Q10:107 (Qebehsenuef) bears a highly fragmented offering formula

**Fig. 4:**
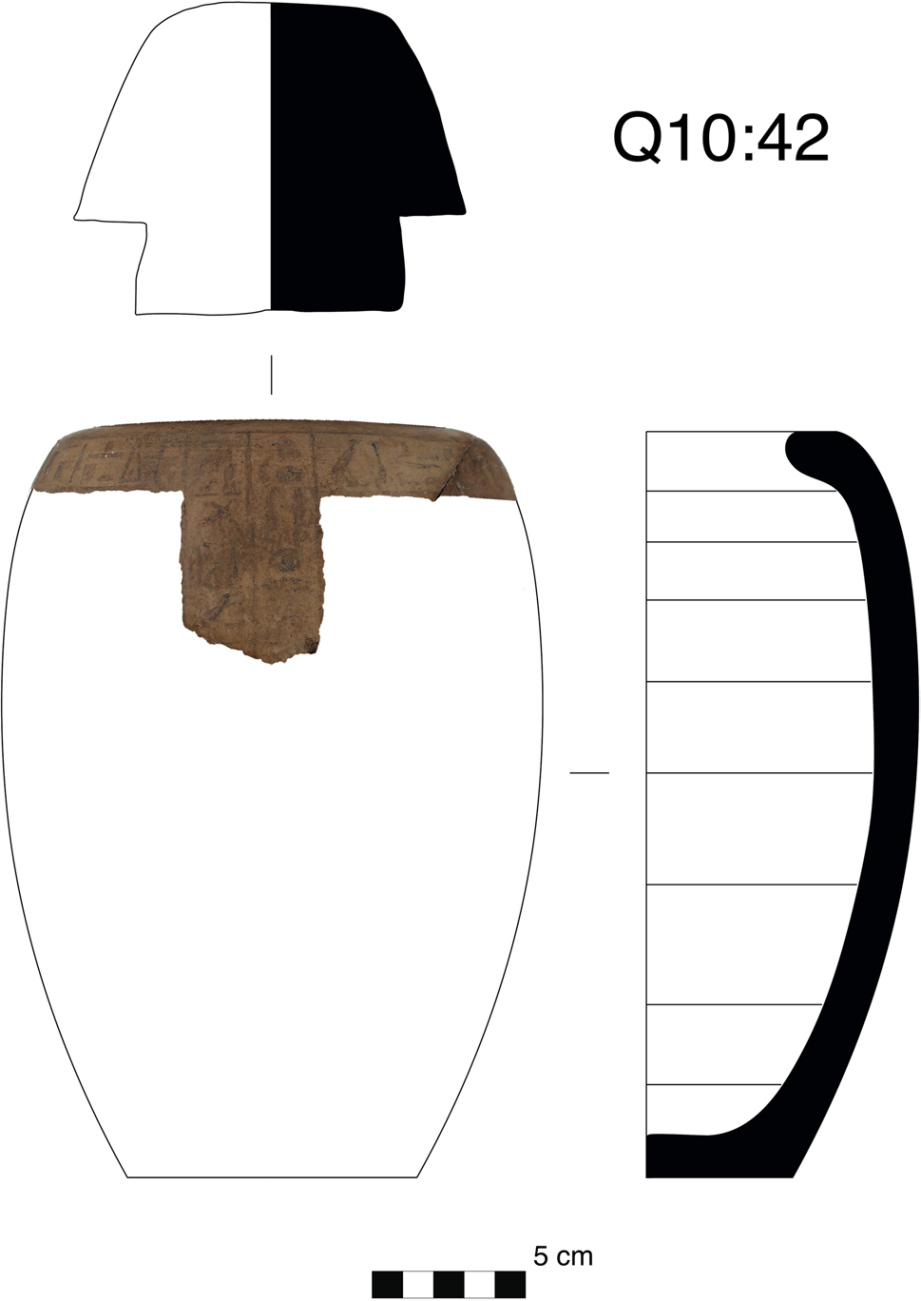
Shape and section of canopic jar Q10:42 (prepared by S. Russo after an original drawing by R. Lemos). Horizontal inscription: “An offering which the king gives to Anubis, the foremost of the sacred tent upon his mountain, may he grant movement in and out of the necropolis […] drink water from the river stream.” Vertical inscription: “Honored by [Hapy], Osiris Paitsy-her, justified” (translated by R. Lemos)

The four canopic jars of Djehutyhotep are typical of the 18th dynasty both in shape and style (Figs. 3 and 4). They were wheel-thrown and made of a hard, compacted pink ware—likely marl clay (Wodzińska 2010: 85)—which indicates they were imported from Egypt to Nubia. The shape consists of a restricted vessel on a flat base terminating in a convergent and concave upper section. The walls are thick, especially on the base, due to the objects’ original function as containers for the deceased’s viscera. The surfaces are uncoated, and the outer surface is decorated with black inscriptions arranged in a “T”-shaped pattern, consisting of a horizontal row below the rim and two vertical columns defining the front of the jars (Holthoer 1977: 78).

The jars’ stoppers are in the shape of a human head wearing a headdress. They were made of an “uncompacted brown ware” with limited straw tempering (Holthoer 1977: 73). This probably indicates Nile clay. The human head-shaped stoppers were modeled on top of a wheel-thrown bowl which worked as a plug. The whole surface of the stoppers was painted red. The observed differences in the fabrics of the jars and the stoppers might suggest that while the jars were imported from Egypt, the stoppers might have been manufactured locally.

The inscriptions on the canopic jars of Djehutyhotep were arranged differently than most of the canopic jars from the same period, on which the protective texts were presented in a square separated by three or four columns (Hayes 1978: 227). On each of Djehutyhoptep’s jars, a vertical inscription arranged in two columns mentions the owner honored by one of the four sons of Horus. This follows the usual norm of contemporaneous Egyptian canopic jars. However, the inscriptions on the canopic jars of Djehutyhotep deviate slightly from the norm as they also consist of offering prayers to Osiris and Anubis by Paitsy-her (Holthoer 1977: 78; Säve-Söderbergh and Troy 1991: 203). The offering formulas on the canopic jars might suggest that, as a whole, the set of canopic jars was conceived differently by Nubian elites in the colony, as in New Kingdom Egypt these objects were not associated with the provision of offerings. The inscriptions also seem to combine hieroglyphs with more cursive versions of the signs, and, occasionally, the scribe seems to have mistaken one sign for another.

The canopic jars of Djehutyhotep were not found in their original depositional context. They were retrieved from the tomb of his brother and successor Amenemhat, on the other bank of the river at Debeira East. All four jars and stoppers were excavated in the corridor leading to the burial chamber, where other objects originally belonging to Djehutyhotep were found, namely, an ivory scribe’s palette bearing his name. It has been suggested that objects owned by Djehutyhotep were moved to the tomb of Amenemhat because Djehutyhotep himself would have been reburied in his brother’s tomb (Säve-Söderbergh and Troy 1991: 76). However, this seems unlikely as both siblings were contemporaneous and evidence from elsewhere points to the reuse and circulation of objects across different burial contexts within the New Kingdom colonial Period (Lemos 2020: 11–12). For instance, two shabtis of Viceroy of Kush Messuy, active in the reign of Merenptah in the late 19th dynasty (Török 2009: 175), were found in tomb S90 at Aniba (Steindorff 1937: plate 44), while another shabti, part of the same group, was retrieved from a rock-cut tomb of the same period at Wadi es-Sebua (Emery and Kirwan 1935: 103–104).

**Fig. 5:**
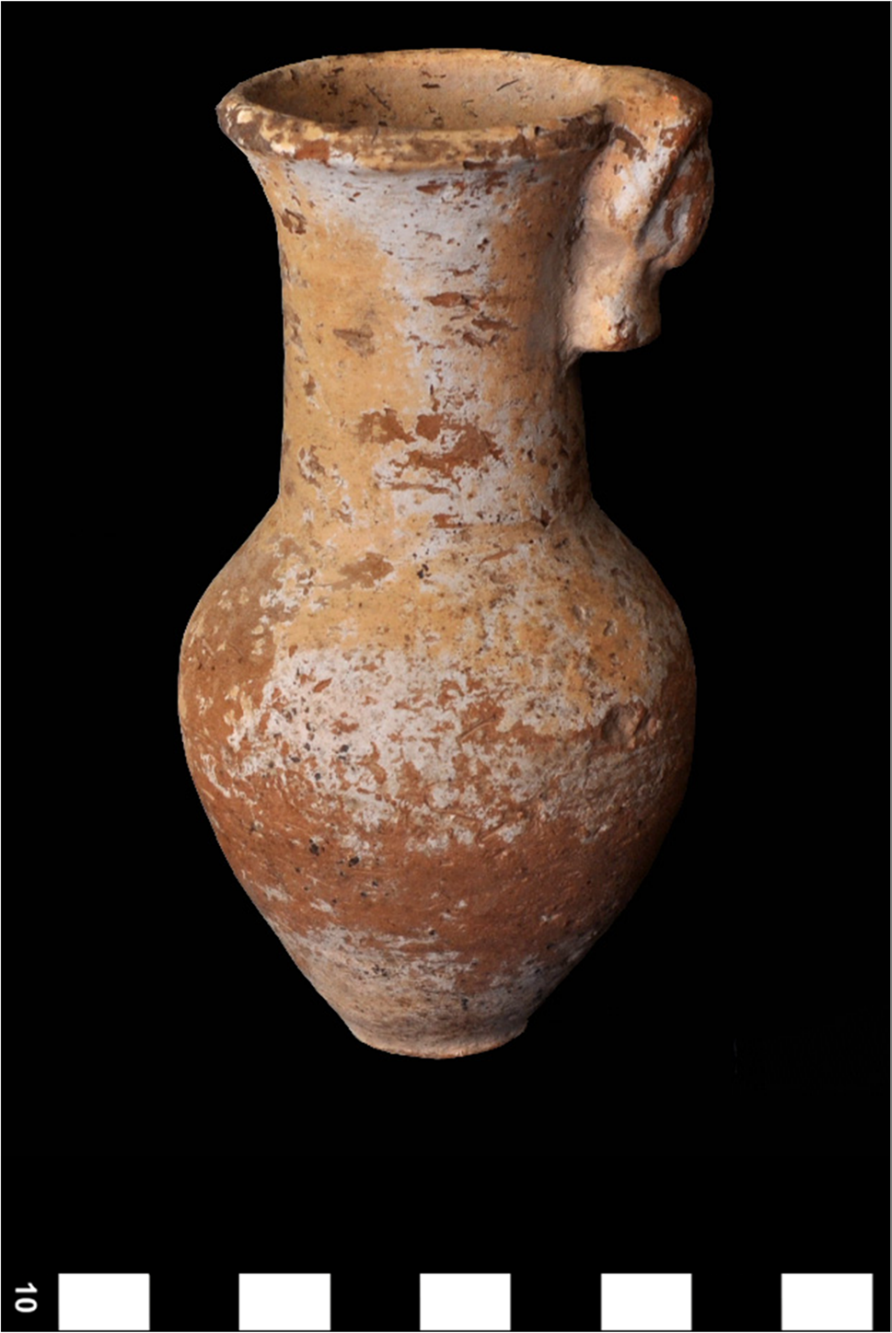
Object SAC5 352/2017 from tomb 26 on Sai Island (Budka 2021: 293; photo by C. Geiger, courtesy of the AcrossBorders Project). A human head seems to have been later attached to the Nile clay jug, transforming a regular vessel into a ritual item which fulfilled the role of a canopic jar, according to how these objects were understood in colonized Nubia

Canopic jars were rare objects in Nubia and restricted to elite contexts (Säve-Söderbergh and Troy 1991: 76; Thill 1996: 87; Lemos 2020: 14). In addition to the Debeira canopic jars, these objects were also excavated at Aniba (Steindorff 1937: 74), Dabod (Reisner 1910: 175), Dakka (Firth 1915: 150; see also Reisner 1910: 341), Dehmit (Reisner 1910: 281), Sai (Minault-Gout and Thill 2012: 170–173, plate 90; Budka 2021: 123–124), Soleb (Schiff Giorgini 1971: 96), and Tombos (Smith and Buzon 2017: 621–622). All canopic jars retrieved in Nubia were made of pottery, with the exception of the ones excavated at Aniba (cf. Näser 2017: 568). Difficulty in accessing these objects led local elites to resort to alternatives, including the use of other types of jars as versions of Egyptian canopic jars. Thill suggests that ordinary pottery shapes could have been used as canopic jars in transitional tombs from the Classic Kerma to the New Kingdom colonial period on Sai Island (Thill 1996: 87; see also Gratien 1978: 220–222). Also on Sai Island, regular small globular jars went through a process of material metamorphosis and became versions of canopic jars, with locally molded clay lumps in the shape of human heads used as stoppers (Lemos and Budka 2021: 407; see also Budka 2021: 123–124). The same might have happened with two small ovoid jugs at Sai (Budka 2021: 124, 205). Small clay heads were added to the rims of the vessels, which would have locally replaced the human-head stoppers of standard canopic jars, then resulting in a locally effective version of these objects (Fig. 5). The jugs were filled with a local recipe of mortuary unguent (Fulcher and Budka 2020), which matches the ritual practice associated with the canopic jars of Djehutyhotep as will be discussed below. However, due to striking material similarities (Smith 2003b: 49; Wodzińska 2010: 59), these jars could have also been former milk jars transformed into local versions of canopic jars. Examples of milk jars have been excavated at the New Kingdom colonial settlement on Sai Island, which reinforces our interpretation (Budka 2016: 92–96).

The presence of canopic jars in New Kingdom colonial Nubia has been considered evidence for the artificial preservation of the body or mummification (Säve-Söderbergh and Troy 1991: 76; cf. Lemos 2018: 29–30). However, to this date, no mummified human remains have been recovered from mortuary contexts of the New Kingdom colonial period in Nubia (Spence 2019: 559). In this paper, we consider mummification as the materialization of an intention to artificially preserve the body for the afterlife. In Egypt, the artificial preservation of the body included a myriad of practices which, together, did not characterize homogeneity, e.g., the wrapping of the body without organ removal (cf. Ikram and Dodson 1998; Taylor 2001). For New Kingdom colonial Nubia, evidence of body wrappings at Tombos has been interpreted as signs of mummification (Buzon 2008: 173). Body wrapping has also been detected at sites such as Soleb and Fadrus (Lemos 2020: 14). Wrapping the body would stabilize body parts, therefore facilitating deposition into the grave. Despite any potential ritual connotation of body wrappings in New Kingdom colonial Nubia, wrapping the body alone does not suggest that the practice was essentially connected to preserving body tissues for the afterlife in the Egyptian way. Moreover, evidence for the use of funerary unguents in colonized Nubia seems to point to variations and different conceptions of funerary practices.

An aspect of the New Kingdom Egyptian mortuary ritual was the pouring of a resinous unguent onto artificially mummified bodies and coffins (Clark et al. 2016). Elements of this ritual have been sporadically detected in colonized Nubia. At Aniba, wrappings associated with two intact, non-mummified interments showed traces of a black resinous liquid (Steindorff 1937: 200). At Amara West, where no mummified human remains have been excavated, examples of coffin fragments and textile fragments showing traces of unguents were found (Fulcher et al. 2020). A further example from Sai demonstrates the use of mortuary unguent with non-mummified human remains. Two dried pieces of a resinous unguent were associated with skeletal remains connected to local versions of canopic jars (Fulcher and Budka 2020: 10).

This evidence suggests that parts of the New Kingdom mortuary ritual reached colonized Nubia, but materialized in different ways, probably following local understandings of the ritual according to varied social experiences within New Kingdom colonial Nubia. Standard canopic jars, such as the set belonging to Djehutyhotep or examples from Aniba, were not associated with mummified bodies, which points towards local meanings attached to these objects other than the preservation of mummified human tissue. Moreover, hitherto no examples of New Kingdom canopic jars containing human tissue have been recovered in Nubia. The unusual inscriptions on the canopic jars of Djehutyhotep would also reinforce such an interpretation. The shape of local versions of canopic jars from Sai alone would also point towards alternative religious conceptions and recreations of dominant mortuary rituals in the colony. Moreover, the restricted character of canopic jars in New Kingdom colonial Nubia suggests that contemporaneous Egyptian mortuary religion only penetrated in Nubia to a limited extent, despite the widespread substitution of indigenous material culture for the dominant patterns of the colonizer. However, as demonstrated by Smith, canopic jars were also restricted to elites in New Kingdom Egypt (Smith 1992: 199; 2003b: 40), not playing a major ritual role among non-elites (Lemos 2018: 29). This would allow us to detect shared social experiences of mortuary religion across Egypt and Nubia: while in Egypt, elites were able to possess canopic jars, which is evidence for the “standard” mummification ritual including the removal of internal organs to be stored in these jars, in Nubia, elites struggled to have access to canopic jars and other Egyptian-style objects, which resulted in the creation of local forms and styles only ritually effective in the colony. In the end, the elite social experience of mortuary religion in Nubia would have been comparable to the experiences of non-elites in Egypt, which produced material innovation in a context of material scarcity (Lemos forthcoming).

Despite the fact that the elements of Egyptian New Kingdom mortuary rituals were adopted in Nubia, the available evidence for mortuary rituals in the colony suggests that mummification was not a part of such rituals. Micro-analysis of canopic jars can help us shed light on local mortuary practices to understand Nubian experiences of dominant mortuary religion. Similarly, since the use of mortuary unguents on bodies and coffins was not necessarily associated with artificial mummification as it was in New Kingdom Egypt, what roles did canopic jars play in Nubian versions of mortuary religion in the New Kingdom colonial period?

## The canopic jars of Djehutyhotep/Paitsy on a micro-scale

### Sampling and methodology

**Fig. 6:**
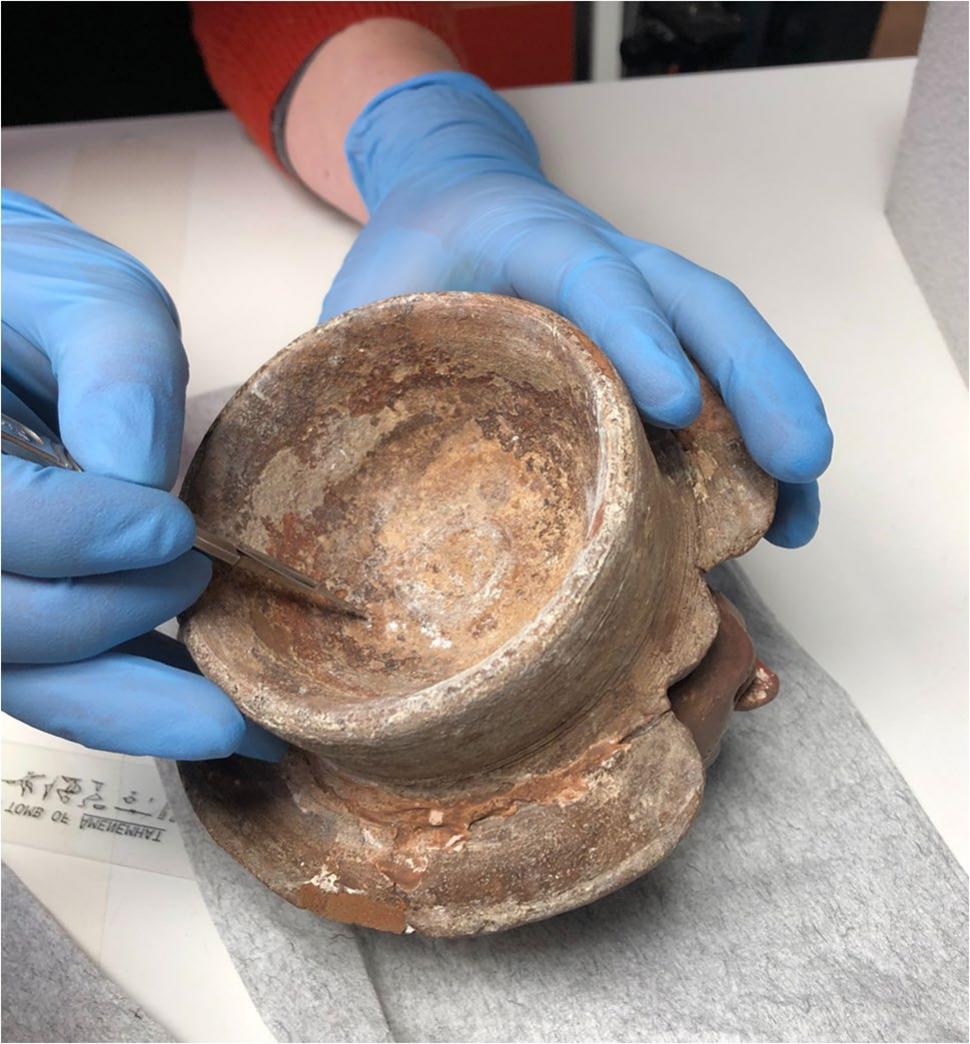
Visible residue on the lid of jar Q10:107 during sampling (photo by R. Lemos, courtesy of the Gustavianum Museum, Uppsala University)

**Fig. 7:**
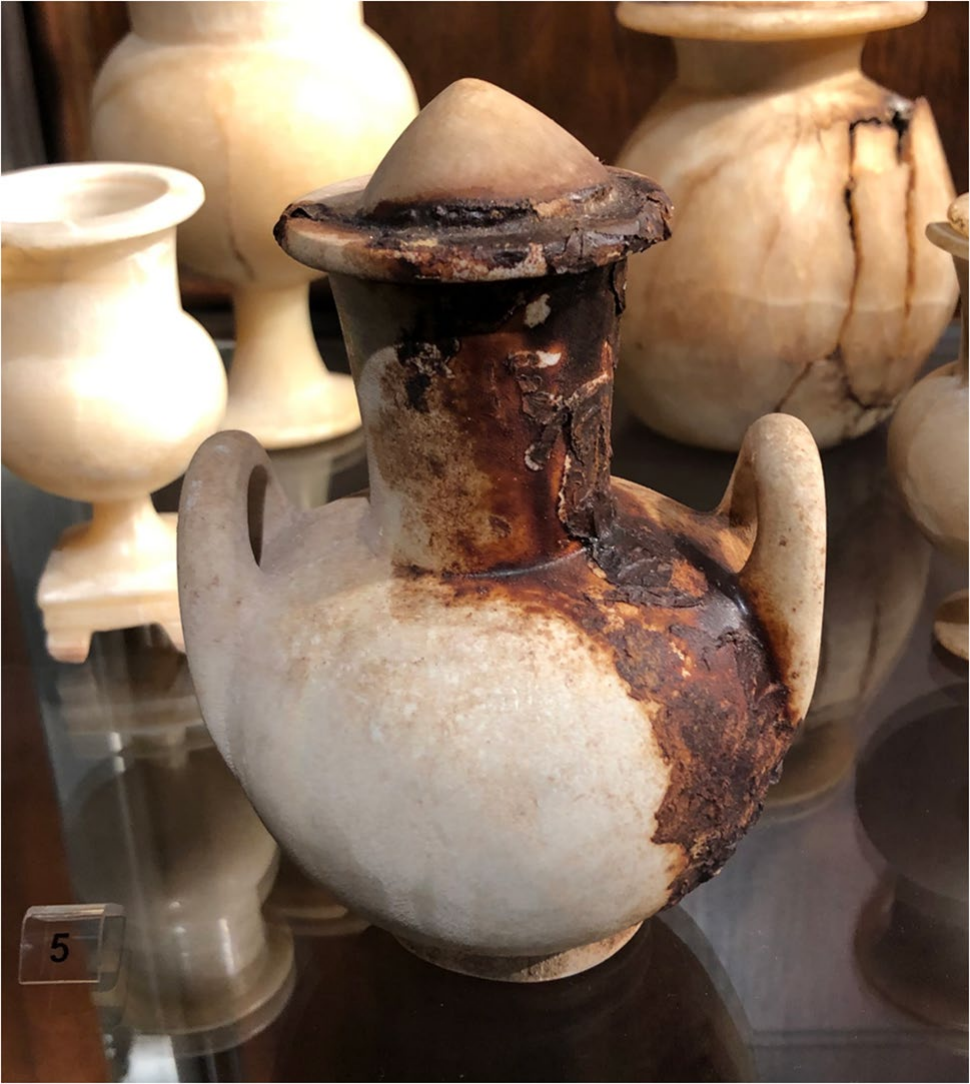
Travertine globular vessel with handles topped by a conical travertine lid (H 15.5 cm, including lid) from Aniba tomb S91, displayed in Leipzig (photo by R. Lemos, courtesy of the Egyptian Museum Georg Steindorff in Leipzig). Stone vessels completely filled with unguent might have played the same ritual role as local versions of canopic jars in the New Kingdom colonial Nubia

To answer the above question, samples of black residue were taken from the three canopic jars of Djehutyhotep kept in the Gustavianum Museum, Uppsala University (Table 1). The samples were taken from both the interior of the jars and the base of the lids. The fact that the same resinous material also remained on the lids demonstrates that the jars were once filled with unguent (Fig. 6). Previously analyzed local versions of canopic jars from Sai were also completely filled with unguent (Fulcher and Budka 2020: 5), as well as stone vessels from Aniba, which might have fulfilled a similar role (Steindorff 1937: 143–144, plate 93, 6 and 8) (Fig. 7).

**Table 1:** Description of samples taken from three of the canopic jars of Djehutyhotep. Inscriptions are faded and sometimes difficult to read

Object	Description	Visible residue?	Samples taken
Q10:42	Hard pink ware (marl clay) tall, gently curving-shaped jar with the maximum diameter at the shoulder. Brown ware (Nile clay?) human head stoppers modeled onto wheel-thrown cups, painted red. Black painted inscription on the jar. Previously broken but reassembled	Yes	Jar and lid
Q10:194	Hard pink ware (marl clay) tall, gently curving-shaped jar with the maximum diameter at the shoulder. Brown ware (Nile clay?) human head stoppers modeled onto wheel-thrown cups, painted red. Black painted inscription on the jar. Previously broken but reassembled	Yes	Jar
Q10:107	Hard pink ware (marl clay) tall, gently curving-shaped jar with the maximum diameter at the shoulder. Brown ware (Nile clay?) human head stoppers modeled onto wheel-thrown cups, painted red. Black painted inscription on the jar. Previously broken but reassembled	Yes	Jar and lid

Samples from the three canopic jars of Djehutyhotep were analyzed using gas chromatography coupled with mass spectrometry (GC–MS) at the British Museum in London. Method A was used to detect lipids (fat, resin): samples were dissolved in 1 ml dichloromethane (DCM) and heated at 40 °C for 2 h, after which the solution was decanted and dried under a stream of nitrogen. This was done 3 times, combining the extracts. Each final dried extract was derivatized using 100 µl silylating reagent N,O-bis(trimethylsilyl)trifuoroacetamide (BSTFA) plus 1% trimethylchlorosilane (TMCS), heated at 70 °C for 1 h. After cooling, samples in BSTFA were auto-injected into the GC. The GC–MS analysis was carried out with an Agilent HP5-MS column (30 m × 0.25 mm, 0.25 µm film thickness) with splitless injection, coupled to an Agilent 5973 MSD. The mass spectrometer was operating in the electron impact (EI) mode at 70 eV and scanning m/z 50 to 750. The oven was set at 60 °C, increasing at 10 °C per minute to 200 °C, and then 3 °C per minute to 325 °C, which was held for 5 min.

Samples were analyzed for bitumen content using method B: samples were dissolved in 1 ml dichloromethane (DCM), and heated at 40 °C for 2 h, after which the solution was decanted and dried under a stream of nitrogen. This was done 3 times, combining the extracts. Then 20 µl DCM and 1 ml hexane were added to the dried extract; the asphaltene fraction precipitated out, and this was left overnight to settle. The soluble solution was decanted and dried under a stream of nitrogen to obtain the maltene fraction. Each maltene fraction was then fractionated using column chromatography. A further 100 µl hexane was added to the maltene fraction. Each was decanted into a glass pipette held upright and plugged with glass wool and half filled with dried silica (chromatography grade 60–120 µm, pre-extracted with DCM/methanol 97:3 (v:v), followed by hexane, then oven dried) to which hexane had been added to exclude moisture. The first fraction was extracted using 3 ml hexane washed through the pipette; the second using 3 ml DCM:hexane 1:3 (v:v); the third using 3 ml DCM:methanol 2:1 (v:v). The elutes were collected and dried in a stream of nitrogen. For analysis, 50 µl of hexane was added to the first fraction, and this was decanted to a micro vial. The GC–MS analysis was carried out with an Agilent HP5-MS column (30 m × 0.25 mm, 0.25 µm film thickness) with splitless injection, coupled to an Agilent 5973 MSD. The mass spectrometer was operating in the electron impact (EI) mode at 70 eV and scanning m/z 50 to 550. The oven was set at 60 °C to 290 °C at 4 °C/min with the final temperature held for 30.5 min. GC–MS analysis was run in two modes: scan and selective ion monitoring (SIM). Acquisition in SIM mode targeted ions: 177, 191, 217, 218, 259.

Samples were analyzed for sugars using Method C based on a published method (Bleton et al. 1996): samples were hydrolyzed by the addition of 500 µl of 0.5 M hydrochloric acid, heated at 80 °C for 20 h. The solution was decanted and dried under nitrogen. Samples were derivatized by the addition of 300 µl Sigma-Sil A (1:3:9 ratio of trimethylchlorosilane (TMCS), hexamethyldisilazane (HMDS) and pyridine) and heated at 80 °C for 2 h. Samples were dried under nitrogen and dissolved in 100 µl hexane in preparation for injection into the GC–MS instrument. Derivatized samples were separated using an Agilent HP5-MS column (30 m × 0.25 mm, 0.25 µm film thickness with 1 m × 0.53 mm retention gap) with splitless injection at 300 °C and 10.1 psi and a purge time of 0.5 min. The oven was set at 40 °C to 130 °C at 9 °C/min, then to 290 °C at 2 °C/min, with the final temperature held for 10 min. Samples were analyzed using an Agilent 5973 MSD operating in the electron impact (EI) mode at 70 eV and scanning over the range m/z 50 to 550. Chromatograms were extracted for ions 204 and 217 to look for the presence of monosaccharides. For all methods, blanks were prepared alongside the samples.

All five samples (three from jars and two from lids, Table 1) from the three canopic jars of Djehutyhotep were analyzed using Masshunter software and the NIST database.

## Results

**Fig. 8:**
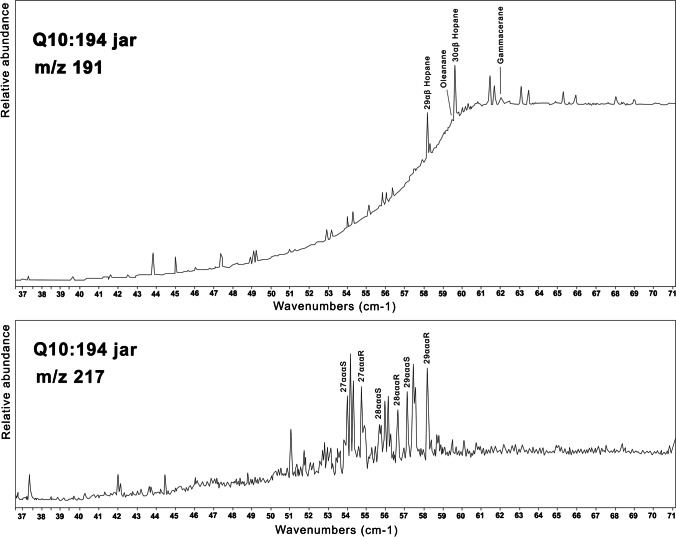
Partial chromatograms (37 to 71 min) for ions 191 (hopanes) and 217 (steranes) from SIM analysis of sample from Q10:194 jar

Chromatograms for lipids analysis were blank or contained very low amounts of fatty acids, consistent with small amounts of contamination from handling etc. (Table 2). All five samples provided evidence for the presence of bitumen, with peaks for hopanes (m/z 191) and steranes (m/z 217). The samples from the Q10:107 jar and Q10:42 jar had only traces of hopanes and steranes, but the samples from the other jars and the lids provided enough data to see that there was a low peak for gammacerane, and the data obtained from the sample from Q10:194 jar showed a small but visible peak for oleanane (Fig. 8). Four of the samples contained monosaccharides (Table 2). The peaks were small and the range of monosaccharides detected varied between samples (Table 3).

**Table 2:** Results from lipid and bitumen analysis of canopic jar samples

Object from which sample was taken	Result—lipids (method A)	Result—bitumen (method B)	Result—sugar (method C)
Q10:107 jar	No	Yes	Yes
Q10:107 lid	No	Yes	Yes
Q10:194 jar	No	Yes	Yes
Q10:42 jar	No	Yes	No
Q10:42 lid	No	Yes	Yes

**Table 3:** Results of sugar analysis—monosaccharides observed in extracted chromatograms (m/z 204 & 217)

Samples	Arabinose	Rhamnose	Fucose	Xylose	Mannose	Galactose
Q10:107 jar	x	x		x	x	x
Q10:107 lid				X		xx
Q10:194 jar	(x)			x	x	x
Q10:42 jar						
Q10:42 lid	x	x	(x)	xx	x	x
Sai SAC5 352	x	x		x	x	x

## From the micro-scale to ritual practice

The residue analyzed from these Nubian canopic jars indicates that the contents consisted of bitumen mixed with a plant gum. It is likely that the gum component is highly degraded, and therefore, it is difficult to interpret, but the presence of mannose suggests that something other than acacia gum was used, as acacia does not contain this monosaccharide (Lluveras-Tenorio et al. 2012). Acacia is now the most commonly used natural gum in Nubia, and it appears from wood remains at archeological sites to have been used as a resource in ancient times, but the environment has changed a great deal over time, and it is not clear which species that may have yielded gum may have been available to the ancient inhabitants (Cartwright & Ryan 2017). However, it has been suggested that the most likely gums available to the ancient Egyptians were acacia, fruit gum, and tragacanth (Newman and Serpico 2000). Acacia contains arabinose and rhamnose, but not fucose and mannose; fruit gum contains mannose but not fucose, and tragacanth contains fucose but not mannose (Lluveras-Tenorio et al. 2012). Xylose is frequently found in the environment, as is glucose (which all the samples contained, but this does not indicate anything of interest). It seems likely that a fruit gum, indicated by the presence of mannose in four samples, was at least one of the gum components used.

Analysis of a large number (*n* = 28) of funerary residues from canopic jars was reported recently (Brockbals et al. 2018). The jars were dated using either radiocarbon dating or stylistic dating, but no archeological information relating to their provenance was reported. They were said to all be from Egypt: six from the Old Kingdom (2686–2160 BCE), three from the Middle Kingdom (2055–1650 BCE), four from the New Kingdom, one from the Third Intermediate Period (1069–664 BCE), and fourteen from the late period (664–332 BCE). The components of the residues were determined using GC–MS; all samples contained fatty acids; nine contained beeswax; most contained terpenoids, which indicate plant extracts or essential oils; and three contained dehydroabietic acid, a marker for conifer resin. No evidence for bitumen was found, but as the authors state, the sample preparation for bitumen was not specifically undertaken, which can lead to bitumen being missed in the analysis (see Łucejko et al. 2017). The ingredients identified in these jars are similar to those used in other ancient Egyptian funerary liquids for mummification and anointing (see below). Another study looked at samples from five “canopic packs” (that had been placed back inside the body cavity) from the Ptolemaic Period; they were also found to contain fatty acids, beeswax, and conifer resin (Tchapla et al. 2004). One of the samples contained monosaccharides, indicating the presence of a plant gum; the study did not apply a specific analytical procedure for bitumen.

Funerary liquids from ancient Egypt, used for mummification, to anoint the wrapped body and coffin, and to coat funerary statues, have been shown to consist of a fairly standard mixture of ingredients. The exact ingredients used in each case can vary, but the list from which they are chosen appears to be consistent. These ingredients always include a fatty material, either plant oil or animal fat, and may also include any of the following: beeswax, *Pistacia* resin, conifer resin or pitch, bitumen (Brettell et al. 2017; Buckley & Evershed 2001; Fulcher et al. 2021; Łucejko et al. 2017; Marković et al. 2020). In some cases, heated *Pistacia* resin appears to be the only ingredient, although evidence for this is currently restricted to coatings on funerary statues (Serpico & White 2001). There may also have been other more volatile components that can no longer be detected.

Most of the bitumen used in the funerary liquids from Egypt has been shown to originate from the Dead Sea (Connan & Dessort 1991; Fulcher et al. 2021; Łucejko et al. 2017; Nissenbaum 1992), although there are exceptions (Harrell & Lewan 2002). There is evidence of the trade of Dead Sea bitumen into Egypt from very early in Egypt’s history (Connan et al. 1992). Dead Sea bitumen has a distinctive pattern of hopanes (m/z 191) and steranes (m/z 217), with no oleanane and high gammacerane (Connan & Dessort 1991; Rullkötter & Nissenbaum 1988). The pattern of hopanes for the canopic jars of Djehutyhotep differs from this Dead Sea pattern. The hopanes for Djehutyhotep show low gammacerane and a visible, although small, peak for oleanane. The peak for the carbon-30 diahopane just to the right of the peak for the 29αβ hopane is much more pronounced than in Dead Sea samples. This indicates that the source of this bitumen was not the Dead Sea. A similar pattern was observed for one sample from the Nubian town of Amara West, PS121 (Fulcher et al. 2020), and for a locally adapted canopic jar from Sai dating to the 18th dynasty (Fulcher and Budka 2020: Fig. 5). This suggests that the bitumen used to produce unguents in New Kingdom colonial Nubia may have traveled across different trade networks, which probably imposed challenges to the access to raw materials, leading to the creation of alternative recipes used in local mortuary contexts.

The mixture of plant gum and bitumen demonstrates that alternative unguent recipes were used in colonized Nubia (Table 3). This is also the case with the results for Djehutyhotep, which further reinforces the identification of a local unguent recipe, potentially connected with local beliefs and practices. Originally, the analyzed canopic jars from Sai were regular jars repurposed to fulfill a specific ritual role, a practice which would probably not suit Egyptian elite conceptions of canopic jars. These repurposed objects were used in combination with a different recipe for unguent, which was used to fill the entirety of the jars in mortuary contexts. The local unguent used might have originated from the difficulties of accessing the raw resources, which probably had to be reached through alternative trade routes to meet local demand for bitumen. The Dead Sea was the source of bitumen used in Egypt. Bitumen from the Dead Sea was only used at Amara West. This demonstrates that some of the bitumen reached Nubia through Egypt, which imposes other social challenges to access. Despite certain variations, the canopic jars of Djehutyhotep follow the Egyptian typology of the time, but their contents consisted of a local unguent recipe, which might suggest alternative practices. The offering formula painted on the Djehutyhotep canopic jars instead of the typical protective spells would further reinforce this idea.

It remains difficult to understand the nature of such practices or where they originate. The local mixtures of alternatively sourced bitumen and plant gum were also used in New Kingdom colonial Nubia in ways similar to contemporary Egyptian ritual practice, namely the pouring of unguent onto coffins and bodies. The differences between Egyptian and Nubian practices include the fact that bodies were not artificially mummified in the Nubian colony, and typical or locally adapted canopic jars were filled with unguent, while in Egypt these jars would contain mummified human tissue. Table 4 summarizes the evidence for the ritual use of mortuary unguents in New Kingdom colonial Nubia.

**Table 4:** Comparison of the use of unguents at various sites in New Kingdom Nubia

Site	Composition	Context
Dabod cemetery 24 (New Kingdom to Roman) (Reisner 1910: 169)	?	Plundering left behind scattered bones and “mummies” covered in unguent (note outdated terminology)
Aniba cemetery S/SA (Steindorff 1937: 200)	?	Poured onto coffins/non-mummified bodies; filling of jars (Fig. 7)
Debeira (Djehutyhotep)	Plant gum; alternatively sourced bitumen	Filling of jars
Saras site 11–Q–62 (Edwards and Mills 2020: 145)	?	Large workshop site; Indigenous architecture including several rooms equipped with tanks and bins; finds include large grindstones, pottery turntables, palettes, and jewelry and “several small fragments of red ochre and several pieces of what may have been bitumen”; probably ground and used as pigment (?)
Amara West (Fulcher et al. 2021)	Plant gum; bitumen from the Dead Sea (same source as bitumen used in Egypt)	Poured onto coffins/non-mummified bodies; ground bitumen used as a pigment; small pieces of bitumen
Sai cemetery SAC5 (Fulcher and Bukda 2020)	Plant gum; alternatively sourced bitumen	Poured onto coffins/non-mummified bodies; filling of jars

Differences in ritual practices in New Kingdom colonial Nubia might point to reshaped versions of the dominant/colonial mortuary religion based on distinctive local experiences of colonization. The existence of a local unguent recipe alone might point towards local conceptions of the afterlife, especially in association with a disregard for the artificial preservation of the body, which would disassociate canopic jars of their Egyptian meaning as containers of mummified viscera. The practice of filling these and other jars with a locally conceptualized unguent can be considered a result of local ritual inputs to the dominant mortuary religion, which could potentially be connected to alternative beliefs. This remains open to be fully investigated and expanding the scope of the available evidence is crucial in that regard. Nonetheless, the offering inscriptions on the canopic jars of Djehutyhotep might point to local religious conceptions of reshaped and repurposed canopic jars, not understood in Nubia as containers for inner organs, but potentially as an offering recipient.

## Conclusions

Canopic jars in colonized Nubia during the New Kingdom were restricted to a few elite individuals who had the means to possess such objects to express cultural affinities with Egyptian colonizers (Smith 2003b: 40; Spence 2019: 560; Lemos 2020: 14). Typical Egyptian canopic jars were adopted by colonial elites in Nubia, as exemplified by the canopic jars of Djehutyhotep. However, creatively adapted versions of these objects, which resulted in complete material transformations, were also consumed among local elites aiming to display cultural affinities with Egypt in an overall context of material scarcity. However, despite colonial elites’ desire to display connections with Egyptian cultural values or even religion, analyses of residues from the inside of these jars suggest variation.

Analysis of samples from the canopic jars of Djehutyhotep adds substance to scholarly interpretations of local creativity and agency in a context of major material impositions during the Egyptian colonization of Nubia in the New Kingdom (Carrano et al. 2009; Smith and Buzon 2017; Smith 2021). In both Lower and Upper Nubia, unguents composed of a mixture of plant gum and bitumen from a different source than bitumen used in Egypt seem to have been a characteristic of colonial mortuary rituals in New Kingdom Nubia. Creatively mixing plant gums and alternatively sourced bitumen points towards socioeconomic issues that are still obscure to scholars today, but might be related to the local difficulty to access resources and desired material culture in the colony, which resulted in various adaptations of typical material shapes and styles (Lemos 2023).

It would be easier to interpret ritual variation as resulting from material challenges to access raw resources and desired funerary objects. Cultural adaptation to difficult social conditions certainly played a major role in reshaping Egyptian mortuary religion locally. However, limiting our interpretations of the available evidence to adaptations of Egyptian dominant ideologies and practices would restrict local—elite—agency and ability to provide cultural inputs to global cultural phenomena. Our knowledge of Nubian religion prior to the New Kingdom is considerably limited as no religious texts were produced by local groups. Despite the fact that it remains difficult to understand the religious nature of distinctive local practices associated with dominant material shapes and ideology, the available evidence nonetheless reveals aspects of local mortuary religion that might contribute to a better understanding of local religious experience outside the shadow of Egyptian textual and iconographic sources.

## Data Availability

All relevant data has been included in the paper. Raw data sets generated during the current study are available from the corresponding author.
